# Impaired Microvascular Response to Muscle Stretching in Chronic Smokers With Type 2 Diabetes

**DOI:** 10.3389/fbioe.2020.00602

**Published:** 2020-06-11

**Authors:** Boon-Hua Low, Yue-Der Lin, Bo-Wen Huang, Taipau Chia, Jian-Guo Bau, Hao-Yu Huang

**Affiliations:** ^1^Division of Endocrinology and Metabolism, Kuang Tien General Hospital, Taichung, Taiwan; ^2^Department of Automatic Control Engineering, Feng Chia University, Taichung, Taiwan; ^3^Bioinformatics and Biomedical Engineering, Feng Chia University, Taichung, Taiwan; ^4^Department of Safety, Health and Environmental Engineering, Hungkuang University, Taichung, Taiwan; ^5^Department of Biomedical Engineering, Hungkuang University, Taichung, Taiwan; ^6^Department of Biomolecular Chemistry, UW School of Medicine and Public Health, University of Wisconsin-Madison, Madison, WI, United States

**Keywords:** cigarette smoking, microcirculation, stretch, reactive hyperemia, laser doppler flowmetry

## Abstract

**Objective:**

Cigarette smoking promotes endothelial dysfunction and is a prominent catalyst for vascular disease. This study employed laser doppler flowmetry (LDF) and spectral analysis to investigate the skin microvascular response to relatively mild stimulus of stretching in diabetic smokers.

**Methods:**

The study population consisted of thirty type 2 diabetic male patients (15 smokers vs. 15 non-smokers) and 15 normal non-smoking subjects. The cutaneous blood flow of the calf at both lower limbs was measured by LDF at a supine position throughout and after muscle stretching by passive dorsiflexion of the ankle.

**Results:**

Following the stretch, post-stretch reactive hyperemia (PSRH) responses were found in all subjects. However, the diabetic non-smokers had relatively higher reactive blood flow than that of the diabetic smokers. The PSRH sustained for a longer time in both diabetic non-smokers and non-diabetic non-smokers in the time domain analysis. By spectral analysis, an observed discrepancy between that of diabetic smokers and diabetic non-smokers was statistically significant. Specifically, the frequency intervals corresponded to a nitric oxide dependent endothelial activity. In addition, an excessive response induced by stretching in frequency intervals of neurogenic activity, when compared with the non-smoking control, was found on diabetic non-smokers.

**Conclusion:**

All subjects expressed the PSRH effect in cutaneous microcirculation after a 10-s stretch stimulus; however, this effect was observed at a significantly lower intensity in chronic smokers with diabetes. The spectral analysis of the skin blood flow signals provides a pathological index for the assessment of the endothelial dysfunction induced by cigarette smoking. Furthermore, the discrepancy of neurovascular function between that of diabetic non-smokers and normal subjects could also be distinguished via the variations of the spectrum related to neurogenic activity.

## Introduction

Diabetes mellitus (DM) is a group of diseases in which the regulatory functions of blood glucose are impaired. The global prevalence of diabetes among adults over 18 years of age has risen from 4.7% in 1980 to 8.5% in 2014 (The Emerging Risk Factors, 2010). The direct and indirect costs due to diabetes have caused a major financial burden on society. According to the World Health Organization, diabetes was the direct cause of 1.6 million deaths and was the 7th leading cause of death in 2016 (WHO, 2016). Among the patients with diabetes, microvascular and macrovascular complications were responsible for most of the morbidity and mortality (Hoerger et al., 2004; Tseng, 2004; Zhou and Byard, 2018), such as peripheral artery disease (PAD), the incidence and extent of which could be predicted by the duration and severity of diabetes mellitus (Adler et al., 2002), with an increase of 1% in glycosylated hemoglobin, the risk of PAD increase by 28%. Impaired circulation on lower extremities easily leads to skin infections, which could develop into ulcers if untreated, thus resulting in amputation, the major cause of mortality in diabetic patients.

Cigarette smoking is a major risk factor of vascular disease. In addition to the mechanisms of nicotine which could induce pancreatic β-cells loss and therefore be responsible for development of insulin resistance or inadequate compensatory insulin secretion responses, which resulted in an increased incidence of diabetes compared with never smokers (Foy et al., 2005; Ott et al., 2010), studies in existing literature have suggested that prolonged smoking habits can induce endothelial dysfunction, an early physiological event in atherosclerosis (Gimbrone and Garcia-Cardena, 2016), throughout the entire circulation system from the conduit arteries (Celermajer et al., 1992, 1993; Zeiher et al., 1995; Gaenzer et al., 2001) to microcirculation (Pellaton et al., 2002; Rossi et al., 2007, 2014; Edvinsson et al., 2008; Avery et al., 2009), and aggravate the micro- and macro-vascular complications of diabetes mellitus (Chang, 2012).

The non-invasive monitoring of the variation of the skin microcirculation induced by various stimuli using laser doppler flowmetry (LDF) combined with spectral analysis of blood perfusion signals is a convenient and commonly used method for assessing the deterioration of tissue perfusion induced by cigarette smoking. Previous studies revealed that smokers had reduction of cutaneous vasodilatory response to warming (Edvinsson et al., 2008; Avery et al., 2009), exercise (Gaenzer et al., 2001), and acetylcholine iontophoresis (Pellaton et al., 2002; Edvinsson et al., 2008). Reactive hyperemia is the transient increase of blood flow that occurs following a brief period of stimuli to the blood vessel. Monitoring the post-occlusive reactive hyperemia (PORH), which is induced by arterial occlusion (Tee et al., 2004; Tikhonova et al., 2010) is another standardized technique for the assessment of microvascular function. Chronic smokers were also noted to display blunted or reduced PORH (Pellaton et al., 2002; Rossi et al., 2007, 2014). However, because PORH is simulated by inflating the cuff on the limb to suprasystolic pressure (usually >200 mmHg) for at least 3 min and then by promptly deflating (Celermajer et al., 1992, 1993; Gaenzer et al., 2001; Pellaton et al., 2002; Rossi et al., 2007, 2014), it is important to note that such stimulus has been reported to cause pain and discomfort to research subjects. Like that of PORH, hyperemia also occurs after stretching. Kruse and Scheuermann (2016) and Kruse et al. (2016) have showed that passive stretch of the plantar flexors created a post-stretch reactive hyperemia (PSRH) on gastrocnemius. With this same principle, the present study attempts to apply a stretch as a milder stimulation method that is less likely to cause discomfort to the subject compared to that of PORH, to investigate if such an increased flow of PSRH would also be observed in cutaneous microcirculation, and to observe the effect of smoking on the deterioration of skin microcirculation in patients with diabetes. It is suggested that chronic smoking can exponentially worsen the deleterious effect of endothelium in diabetic patients, and their microvascular dilation response to stretch can also be reduced. To test this hypothesis, the cutaneous blood flow of the calf was recorded by use of LDF on both type 2 diabetic smokers and non-smokers. This data was consistently monitored both during and after passive dorsiflexion of the ankle. In addition to the smoking diabetic group and non-smoking diabetic group, we also recruited a non-diabetic and non-smoking healthy control group to confirm the microvascular functions of the non-smoking diabetic subjects.

## Materials and Methods

### Participants

The primary objective of the study was to examine the influence of cigarette smoking on the microcirculatory function in the lower extremities of diabetic patients. Because type 2 diabetes, accounting for more than 90% of all diabetic cases, is the most common, and the prevalence of type 2 diabetes is steadily increasing worldwide, only type 2 diabetic patients (both smoking and non-smoking) were recruited in this study. Thirty type 2 diabetic male patients were recruited. Within this group, 15 subjects are current cigarette smokers (diabetic smoker group), while another 15 subjects have never smoked (diabetic non-smoker group). The lifetime exposure to cigarette smoking for the smoking subjects is evaluated comparatively by pack-years. Pack-years are determined by multiplying the packs of cigarettes smoked per day, by the number of years the person has smoked. To avoid confounding variables caused by DM complications, only moderately, well-controlled, and relatively new diabetic patients were recruited for the present study. The criteria for the study’s diabetic patients must be free from: heart failure, cerebrovascular accident (CVA), renal failure, diabetic foot disorders, anemia, ankle-brachial index (ABI) less than 0.9, and a body mass index (BMI) larger than 33. Furthermore, fifteen apparently healthy volunteers were recruited as normal subjects. Exclusion criteria for normal subjects were a family history of diabetes, and passive smoking exposure. All participants were informed about the objectives and procedures of the study and signed the informed consent before trial. This investigation was approved and monitored by the ethics committee of Kuang Tien General Hospital in Taichung city (approval number: ktgh 10519).

### Study Design

Because the subject lay down from the upright position, superficial tissue perfusion may be affected due to venous return. According to our previous experience, it takes about 30 min or more for LDF skin perfusion signals to reach a stable value (the variation of microcirculatory signals is less than 5%) when the microcirculatory signals were monitored on lower extremities (It takes much shorter time to reach a stable perfusion signals if the measurement is conducted on upper extremities). Therefore, all participants were asked to relax on a bed at the supine position for at least 40 min before test. In this same day, participants were also asked to refrain from exercise, caffeine, and alcohol consumption for at least 6 h prior to the examination. The skin microcirculation on the calf around the maximal circumference of the gastrocnemius was measured in three separate periods. To begin with, a 200-s measurement was conducted at the baseline, the second measurement was then taken in the muscle stretching period, and the last measurement of 220 s was conducted 2 s after the stretched muscle returned to its relaxed posture. Therefore, both the microvascular perfusions in stretch status and in post-stretch status (recovery period) could be obtained. The conventional occlusion time for PORH experiments is at least 3 min (Raitakari and Celermajer, 2000; Tee et al., 2004; Tikhonova et al., 2010), whereas the stretching time for PSRH is 4 min (Kruse and Scheuermann, 2016; Kruse et al., 2016). Contrary to this common practice, we adopted the PSRH following a shorter period, 10 s, of passive stretch in this report (Cipriani et al., 2003). The skin of the monitored site was stretched by passive dorsiflexion. The passive dorsiflexion was applied by the operator pushing the subject’s feet in the direction of dorsiflexion to the maximum range of motion (ROM) of the ankle joint. As soon as the maximum ROM was achieved, the pushing force was recorded. The ankle joint was then held in this position for 10 s and the microcirculatory signal was recorded simultaneously in the 10-s stretching period. Muscle strength has been found to be highly correlated with body weight (Bohannon, 1997). We assessed the intensity of the pushing force in passive dorsiflexion by the normalized force, which is defined as the stretching force divided by body weight. The trial was conducted on both legs of the participants. From this experimental procedure, there were collectively 30 measurements for each group in this study.

### Instrumentation

The microcirculatory function can be evaluated by several techniques. LDF offers the advantages of non-invasive and real-time measurements. Therefore, it is the most adopted technique, especially in the study of skin microcirculation. LDF includes two optical fibers; one of the fibers transmits the laser light to the target, whereas the other one receives the light reflected back from the target. If the laser light is refracted by the moving blood cells, the light frequency will be shifted. This phenomenon is termed as the Doppler shift. The frequency shift is proportional to the velocity of moving cells.

The blood flow can be obtained by analyzing the intensity and Doppler shift of the refracted light. Unlike most studies in which the conventional low-laser-power LDF were utilized, this study preferred a high-laser-power LDF (VMS-LDF1-HP, Moor Instruments, United Kingdom) with a 785 nm, 20 mW laser (Class 3R per IEC 60825-1:2007), and a wider separation (4.0 mm) non-invasive skin probe (VP1-V2-HP), because the laser can go deeper and test the arterioles which are at a depth of about 1–2 mm and regulate the skin perfusion via endothelia activity and myogenic activity of vascular smooth muscle. According to Clough’s study, the sampling depth could reach a distance greater than 1.4 mm (Clough et al., 2009), and less than 1 mm with a fiber separation of 0.5 mm for the case of low-laser-power LDF. Calibration of the probes was performed using aqueous suspension of polystyrene latex particles before tests. The standard value was provided from the Brownian motion in the aqueous suspension. The LDF signals were acquired with a sampling frequency of 40 Hz and were analyzed by software provided by the manufacturer (moorVMS-PC, version 3.1).

### Data Analysis

The baseline blood flow is defined as the average value of the 200-s baseline segment. In addition, the mean value of the 10-s stretching segment is defined as the stretching blood flow. Finally, the blood flow of the 220-s post-stretch segment was averaged every 10 s. In addition to performing time domain analysis, the spectrum of the blood flow signals was also analyzed to investigate the mechanisms of microvascular regulations. It has been reported that the LDF signal in the frequency interval of 0.005 and 0.15 Hz is influenced by different physiological activities (Kvernmo et al., 1998, 1999). The frequency interval from 0.005 to 0.0095 Hz is influenced by NO-independent endothelial activity; that from 0.0095 to 0.02 Hz to NO-dependent endothelial activity, the interval from 0.02 to 0.06 Hz to the neurogenic activity of the vessel wall, and that from 0.06 to 0.15 Hz to the myogenic activity of vascular smooth muscle. To cover the low-frequency information to 0.005 Hz, the signal for spectral analysis must be a minimum of 200 s in length. Each 220-s post-stretch signal was cut to three segments of 200-s length to trace the variation of the spectrum along the post-stretch period. The first segment ranged from 0 to 200th second of the 220-s signal, which is the signal just after the stretch cessation and is denoted as 0-s in our later discussion. The second segment was from 10th to 210th second of the 220-s signal, that is the segment with a 10-s delay from the first signal segment and is denoted as a 10-s delay. The third segment was from 20th to 220th second and is named as 20-s delay in later content. The spectra of the 200-s baseline, 0-s, 10-s delay, and 20-s delay were calculated. The spectral analysis of the LDF signal was conducted by complex Morlet wavelet transform (by Matlab 2017a, the MathWorks, Natick, MA, United States). Before calculating the spectrum of each 200-s signal, the mean value was subtracted to remove the DC component. Finally, the integrals for the following frequency intervals (I) 0.005–0.0095 Hz, (II) 0.0095–0.02 Hz, (III) 0.02–0.06 Hz, and (IV) 0.06–0.15 Hz were derived via the trapezoidal method, respectively.

### Statistics

Descriptive statistics data are expressed as mean ± SD (standard deviation) unless otherwise stated. In the time domain analysis, in order to confirm if the increased flow of PSRH would be observed in cutaneous microcirculation, a one-way repeated measurement analysis of variance (ANOVA) was conducted within the three groups, respectively, to compare mean values of the microcirculatory blood flow for baseline and each 10-s segments during PSRH period. Furthermore, an independent sample one-way ANOVA was used to compare the normalized blood flows between the three groups for baseline and each 10-s segments during PSRH period. A Bonferroni’s range test was used for *post hoc* multiple comparisons. In the frequency domain analysis, an independent sample one-way ANOVA was used to compare the integral of the wavelet spectral amplitude of the blood flow signals between the three groups for the basal and post-stretch (0-s, 10-s delay, and 20-s delay) in four frequency intervals: 0.005–0.095, 0.095–0.02, 0.02–0.06, and 0.06–0.15 Hz, respectively. In addition, a one-way repeated measurement ANOVA was conducted within the three groups, respectively, to compare the integral of the wavelet spectral amplitude for baseline and three 200-s PSRH stages (0-s, 10-s delay, and 20-s delay). Statistical analysis was performed using SPSS (release 22.0, SPSS Inc., Chicago, IL, United States). Statistical significance was set at *p* < 0.05.

## Results

### Participants

The clinical characteristics of the diabetic non-smokers, diabetic smokers, and the normal subjects are shown in Table 1. The diabetic subjects had higher BMI than normal subjects (*p* < 0.05). The heart rates of the diabetic smokers were higher than that of the diabetic non-smokers and the normal subjects (*p* < 0.05). The diabetic smokers displayed lower ABI than diabetic non-smokers in both legs. The difference in the biochemical parameters between smokers and non-smokers was not significant.

**TABLE 1:** The clinical characteristics of normal subjects, the diabetic non-smokers, and diabetic smokers, mean ± SD.

	Normal subjects (*n* = 15)	Diabetic non-smokers (*n* = 15)	Diabetic smokers (*n* = 15)
Sex (M/F)	15/0	15/0	15/0
Age (years)	52.7 ± 5.6	56.3 ± 8.6	56.9 ± 9.1
Smoking history (pack-years)	0	0	39.9 ± 13.5
Duration of DM (years)	0	7.3 ± 3.3	6.9 ± 3.3
BMI (kg/m^2^)	23.1 ± 1.7*^#^	25.6 ± 3.1	25.8 ± 4.2
Blood pressure			
Systolic (mmHg)	119.9 ± 9.5	127.1 ± 11.3	127.5 ± 11.5
Diastolic (mmHg)	74.2 ± 8.6	73.5 ± 9.0	74.1 ± 7.5
Heart rate (beat/min)	73.4 ± 9.9^#^	77.6 ± 8.7^$^	85.1 ± 6.7
HbA_1c_ (%)	–	7.3 ± 0.5	7.4 ± 1.0
Serum creatinine (mg/dl)	–	0.91 ± 0.15	0.89 ± 0.13
Hb (g/dl)	–	14.9 ± 0.8	15.4 ± 1.3
eGFR (ml/min/1.73 m^2^)	–	95.4 ± 20.1	97.3 ± 19.4
ABI (right leg)	–	1.14 ± 0.05^$^	1.08 ± 0.08
ABI (left leg)	–	1.12 ± 0.06	1.08 ± 0.07
Stretching force/weight (RL) (%)	21.7 ± 3.1	22.3 ± 3.6	22.1 ± 4.8
Stretching force/weight (LL) (%)	21.5 ± 3.1	22.1 ± 3.8	22.0 ± 4.7

DM, diabetes mellitus; BMI, body mass index; HbA_1c_, hemoglobin A_1c_; Hb, hemoglobin; eGFR, estimated glomerular filtration rate; ABI, ankle brachial index; RL, right leg; LL, left leg; N, normal subjects; DN, diabetic non-smokers; DS, diabetic smokers. *N vs. DN: p < 0.05 ^#^N vs. DS: p < 0.05 ^$^DN vs. DS: p < 0.05.

### Microcirculation During and After Muscle Stretch

**FIGURE 1:**
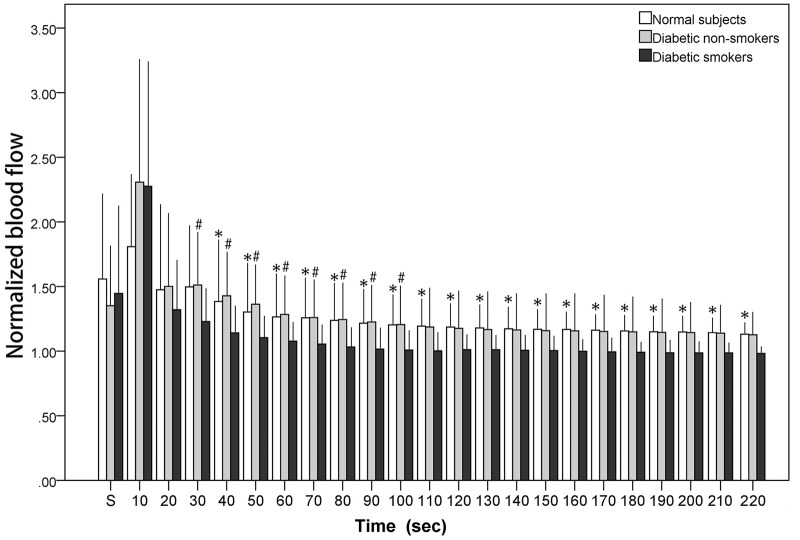
The normalized blood flow of the calf skin averaged in each 10-s segment during stretch (“s”) and immediately after the stretch. # diabetic smokers vs. diabetic non-smokers (*p* < 0.01). * diabetic smokers vs. normal subjects (*p* < 0.01).

Figure 1 illustrates the blood flow during and after stretch. The *x*-axis denotes time while the *y*-axis expresses the normalized blood flow. All the values exhibited in Figure 1 were normalized (divided by personal baseline blood flow) to adjust to individual differences accordingly. Ultimately, if the value is greater than one, this implies that the blood flow was higher than the baseline value. The normalized stretching blood flow was noted with a mark “s.” Figure 1 depicts blood flow elevating drastically within the first 10 s followed by a gradual decrease. The present study revealed reactive hyperemia in cutaneous microcirculation also occurs after stretching in all subjects, including the normal subjects and the diabetic subjects (both diabetic non-smokers and smokers). However, PSRH response was found to be lower in diabetic smokers when compared with the diabetic non-smokers. Although there was no notable differences statistically between groups during the 10-s stretching period and the first 20 s after the stretch, the significant differences between diabetic smokers and diabetic non-smokers became comparatively more observable from the 30-s segment on to the 100-s segment after the stretch (*p* < 0.01). On the other hand, the difference between the diabetic non-smokers and the normal subjects was indistinguishable. The observed PSRH sustained for longer up to the 220-s segment for the normal subjects; to the 210-s segment for the diabetic non-smokers; only to the 90-s segment for the diabetic smokers.

**FIGURE 2:**
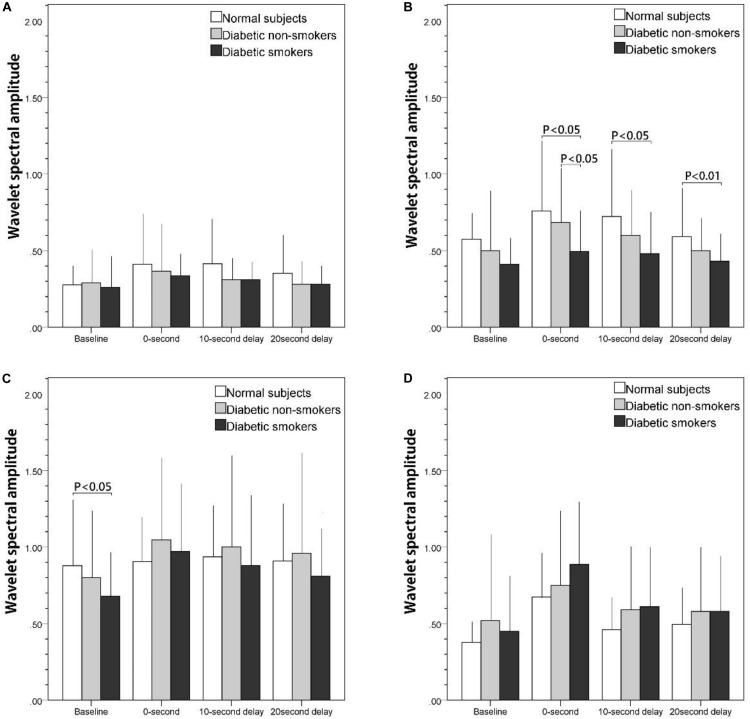
The effect of stretch on skin blood flow motion expressed as the integral of the wavelet spectral amplitude of basal and post-stretch (0-s, 10-s delay, and 20-s delay) in four frequency intervals: **(A)** 0.005–0.095 Hz, **(B)** 0.095–0.02 Hz, **(C)** 0.02–0.06 Hz, **(D)** 0.06–0.15 Hz.

Figures 2A–D illustrate the wavelet spectrum of the microcirculatory perfusion signal during baseline and three PSRH stages in four particular frequency intervals: (a) from 0.005 to 0.0095 Hz, (b) from 0.0095 to 0.02 Hz, (c) from 0.02 to 0.06 Hz, and (d) from 0.06 to 0.15 Hz, respectively. The wavelet spectral amplitude of PSRH response in Figure 2A (NO-independent endothelial activity) and 2b (NO-dependent endothelial activity) show a similar trend, the intensity increased in the first 200-s signal segment (0-s) and then decreased gradually. Compared with baseline, the increase of the 0-s signal segment was statistically significant for the normal subjects and the diabetic non-smokers (*p* < 0.05, not shown on figure), while the increase was not prominent for the diabetic smokers in the frequency interval of NO-dependent endothelial activity (Figure 2B). Therefore, the difference between the normal and diabetic smokers, and the difference between diabetic non-smokers and diabetic smokers was statistically significant in the 0-s segment (*p* < 0.05). Figure 2C shows the intensity of the spectrum of the neurogenic activity. They also reached the maximum value in the 0-s segment (*p* < 0.05) and then decreased gradually in the next two periods for diabetic subjects (both non-smokers and smokers), while there was almost no neurogenic response for the normal subjects. Compared with the normal subjects, the intensity of neurogenic activity in baseline was significantly lower for smokers (*p* < 0.05). The spectrum of vascular myogenic activity increased only in the 0-s segment, and then reverted to the near baseline value in the following 10-s-delay and 20-s-delay periods (Figure 2D).

## Discussion

The primary findings of the present study are that reactive hyperemia occurs in the cutaneous microcirculation of the calf after a brief period of passive muscle stretching. The normal subjects and diabetic non-smokers had relatively higher reactive blood flow than diabetic smokers after the muscle stretching. In addition, the wavelet spectrum of the PSRH signal revealed that the prominent difference between smokers and non-smokers (both the diabetic ones and the normal subjects) was chiefly related to the frequency intervals corresponding to NO-dependent endothelial activity.

Reactive hyperemia is the increase in blood flow when blood vessels are stimulated. PORH induced by total obstruction of blood vessels (therefore causing distal tissue ischemia) occurs when the occlusion of arteries is released. In contrast to the occlusion of arteries, the present research studied the reactive hyperemia induced by stretch. According to Kindig and Poole’s animal study of the effects of stretching on blood flow with different stretching ranges, the effects of stretching on the vascular system should be closely related to the intensity of the stretch. The higher the intensity of the stretch, the more restriction there is on blood flow. Their findings illustrate that the arteriolar diameter does not decrease as the sarcomere length increases to the upper extreme of normal physiological activity (Kindig and Poole, 2001). However, more intense stretching beyond the extreme ranges of physiological activity could potentially result in the decrease of the luminal diameter (Poole et al., 1997) and reduce blood perfusion accordingly. The intensity-dependent effect of the stretch on circulation was also investigated by Kruse and Scheuermann (2016). They measured the microvascular blood volume with near-infrared spectroscopy (NIRS) and discovered that if the intensity of the applied stretch was higher, both the sum of deoxygenated and oxygenated forms (i.e., total blood volume) and the deoxygenated form itself, ([HHb], the sum of the concentration of deoxygenated hemoglobin and myoglobin), was also higher. According to their study, the 240-s low-intensity muscle stretch prompted an initial increase (about 60 s) in [HHb]. This was then followed by a steady state, which was some 20 ∼ 30% of the plateau (i.e., the maximal value) recorded during the occlusion with 250 mmHg. Ultimately, this signifies that the venous return and microvascular perfusion of the stretched area were only partially impeded, resulting in lower accumulation of [HHb]. Furthermore, [HHb] value increased along all stretching periods and reached a value greater than 50% of the maximal value during the 240-s moderate-intensity stretch. This suggests that the microvascular perfusion and venous return were impeded even further. Consequently, the accumulation of [HHb] was found to be even higher. The stretch intensity applied in the present study was only about 80% of the weakest stretch intensity used in Nicholas’s investigation. In our previous pilot studies of healthy subjects in their 20 s, the skin was induced a reactive hyperemia and the effect sustained for longer up to 220 s if the ratio of stretching force to body weight was set to 25.8% ± 1.3%; to 40 s if the ratio was set to 20.1% ± 1.0%, less than 30 s if the ratio was set to 14.3% ± 0.7%. To balance the discomfort and sufficient response of the reactive hyperemia, the applied stretching intensity was about 22% in this study, and the cutaneous PSRH was found after stretch as illustrated in Figure 1. It reveals that skin PSRH is another prominent reaction like PORH despite that the stretch intensity being applied is relatively mild and only 10 s in duration. Compared with Kruse’s study, in which calf muscle extension was also performed by passive dorsiflexion (Kruse et al., 2016), popliteal artery blood flow monitored by Doppler ultrasound consistently increased for 15 s immediately after the 4-min stretch, whereas microvascular blood volume measured by NIRS was enhanced for up to 10 min. It is important to note that our study utilizes the cutaneous microcirculation approach to understand the stretch-induced hyperemia on skin, while Kruse studied the hyperemia occurring in deeper muscle tissues.

Figure 1 shows that there was no significant variation observed between the diabetic non-smokers and diabetic smokers during the stretching (as marked on the first column “S”) and first 20 s after stretching. However, a higher PSRH response was later discovered in the diabetic non-smokers than in the diabetic smokers, specifically from the 20th second after stretching. These results suggest that cigarette smoking can induce a reduced peripheral microvascular response to stretch. On the other hand, there was no significant variation observed between the diabetic non-smokers and the normal subjects. Our findings in PSRH are congruent with the conclusions of the investigations studied among other stimuli about the effects of cigarette smoking. Rossi studied LDF signals obtained from forearm skin during the PORH period. He found that the post-ischemic percent increased from baseline was significantly lower in smokers compared to that of non-smokers (Rossi et al., 2014). Similarly, Pellaton et al. (2002) examining PORH via laser Doppler imaging also observed that the vasodilatory functions in the skin were reduced for older cigarette smokers. In studies using a thermal stimulus on the forearm skin (Edvinsson et al., 2008; Avery et al., 2009), there was also a noted reduction in the warming-induced increase of microvascular blood flow in smokers. Edvinsson et al. (2008) and Pellaton et al. (2002) studied the skin microvascular response to acetylcholine (endothelium-dependent vasodilator) and sodium nitroprusside (endothelium-independent dilator). Likewise, our results revealed that the vasodilatory functions in the skin’s microvasculature of smokers were blunted.

Compared with the results shown in the time domain (Figure 1), in which PSRH responses of diabetic smokers were lower than that of the diabetic non-smokers and the normal subjects, the wavelet spectral amplitude of the PSRH response was also lower in smokers in the frequency interval related to NO-dependent endothelial activity (Figure 2B). Specifically, the discrepancies were statistically significant during the 0-s signal segment compared with the diabetic non-smokers, and during all the 220 s in the post-stretch period compared with the normal subjects. Our observations suggested that an impaired endothelial response to stretch was associated with cigarette smoking. The presented results agree with previous research findings. Primarily, the post-ischemic increase concerning total wavelet spectral density of microcirculatory flow motion in the frequency interval related to endothelial activity was absent in smokers (Rossi et al., 2007). In the investigation using thermal stimulus on the forearm skin, the attenuation was found to be associated with a reduction in spectral amplitude around the frequency considered to correspond to endothelial activity in smokers (Avery et al., 2009). Therefore, they suggest that endothelial microvascular dysfunction was associated with chronic smoking habit. On the other hand, Celermajer studied the endothelium-dependent dilatation of conduit arteries by measuring the diameter increase of the arteries (Celermajer et al., 1992). The flow-mediated dilatation (FMD) was found to be much lower in smokers. We propose that when blood vessel graduates from a stretch to relaxed position, blood vessels apply a shear force on blood and therefore induces a relative motion between blood vessel and blood. In other words, the PSRH phenomena are similar to that of the FMD effect in which vessel dilatation occurs if the blood flow suddenly increases and causes a relative motion between blood vessels and blood. The effects of stretch could be found at both the conduit artery and arterioles of skin.

Another point of consideration for the PSRH effect is to understand the influence of longitudinal tethering on the blood vessels tension. If the blood vessels are stretched in an axial (longitudinal) direction, the vessels could potentially contract in the radial direction, perpendicular to the stretch force, the so-called Poisson effect. To maintain a stable blood supply, the vessel wall must dilate to sustain a stable vessel diameter. Thus, as the stretch force is released, the smooth muscle will not immediately contract. Instead, the dilated arterioles will undergo a flooding of blood into the blood vessels, resulting in a large blood flow pulse. If the stretch could induce vascular dilatation of the upstream feeding blood vessels, the flow pulse could be even higher. This flow pulse will disappear as soon as the smooth muscle returns to its original vessel tone. Thus, we suggest that it only takes 10 s for the smooth muscle to recover back to normal tension. This is the reason why the increase of the spectrum in vascular myogenic activity appears only in the 0-s segment and then disappears during the 10-s-delay and 20-s-delay periods (Figure 2D).

Generally, an ABI value of <0.9 indicates lower extremity PAD (Aboyans et al., 2012). All patients in our study had a normal ABI value between 0.9 and 1.4. However, the mean ABI of the diabetic smokers was significantly lower than that of the diabetic non-smokers. Our results were consistent with the study of Syvänen et al. (2007), where the ABI of smokers in the general population, without any suspected vascular disease, was 0.05 lower than the ABI of non-smokers. This finding implies that the impact of smoking on vessels started well before clinical PAD developed. In conclusion, the observations regarding microvascular response to stretching, results from ABI, and previous works unanimously support the fact that cigarette smoking will cause vascular dysfunction regardless of vessels in systemic circulation or microvascular vessels of diabetic patients.

Diabetic autonomic neuropathy (DAN) is announced as another serious and common complication of diabetes. Both neurovascular-dependent and neurovascular-independent (metabolic or autoimmune damages) factors have been implicated in this pathogenic process (Vinik et al., 2003). When only moderately severe diabetic patients (the mean duration of diabetes was 7.2 years and the mean glycosylated hemoglobin was 7.37%) were recruited in the present study, there was no significant variations of PSRH observed between the diabetic non-smokers and the normal subjects in time domain analysis (Figure 1). On the other hand, spectral analysis will provide further information independent of the endothelia regulation. When we compared the variations of the spectrum of the frequency interval related to the neurogenic activity along the three post-stretch period (0-s, 10-s delay, and 20-s delay) with the baseline (Figure 2C), there were a significant response induced by the stretch stimulus in the diabetic non-smokers, while there was none in the normal subjects. This result indirectly indicates that the response induced by stretching in the frequency interval 0.02–0.06 Hz could provide as an index of the neurogenic activity for the diagnosis of the early stage of DAN independent of the endothelia regulation of vessel wall. In Mima’s study, DAN has been proved to be a significant determinant of skin transcutaneous oxygen tension independent of arterial stenosis and sensory neuropathy in patients with PAD (Hsiu et al., 2010).

The present study was conducted on male subjects; the influence of cigarette smoking on the microvascular response in diabetic females needs further study. The majority of the study population were heavy smokers with a mean smoking pack-year of 40. Results of the study may not be applied to smokers of less intensity. Furthermore, we suggest that the PSRH responses could vary with different stretching forces. A well-controlled stretch intensity, which could be accomplished by regulating the stretching length ratio or constraining normalized stretching force precisely, will reduce the variation of measurement and therefore improve the diagnostic power of PSRH technology for clinical study in future investigations.

## Conclusion

The present study demonstrated that within a 10-s stretch performed by passive dorsiflexion of the ankle, cutaneous microvascular hyperemia (PSRH effect) on calf could be observed unanimously. However, the post-stretch hyperemia response sustained significantly longer in non-smokers. The prominent differences in the spectrum of the perfusion signals between smokers and non-smokers appeared in the frequency intervals from 0.0095 to 0.02 Hz. The signals in this interval are related to NO-dependent endothelial activity. Conclusively, the PSRH effect can be used as an effective tool for the study of blood vessel regulation.

## Data Availability Statement

The datasets generated for this study are available on request to the corresponding author.

## Ethics Statement

The studies involving human participants were reviewed and approved by Kuang Tien General Hospital (approval number: ktgh 10519). The patients/participants provided their written informed consent to participate in this study.

## Author Contributions

J-GB, Y-DL, and B-HL: conceptualization. J-GB: supervision. Y-DL: software. J-GB and B-WH: methodology and investigation. J-GB, B-HL, TC, and H-YH: writing. TC: project administration.

## Conflict of Interest

The authors declare that the research was conducted in the absence of any commercial or financial relationships that could be construed as a potential conflict of interest. The reviewer JM and handling Editor declared their shared affiliation at the time of review.
